# Mucosal exposure to cockroach extract induces allergic sensitization and allergic airway inflammation

**DOI:** 10.1186/1710-1492-7-22

**Published:** 2011-12-14

**Authors:** Narcy G Arizmendi, Melanie Abel, Lakshmi Puttagunta, Muhammad Asaduzzaman, Courtney Davidson, Khalil Karimi, Paul Forsythe, Harissios Vliagoftis

**Affiliations:** 1 Pulmonary Research Group, Department of Medicine, University of Alberta, Edmonton, AB Canada; 2 Department of Laboratory Medicine and Pathology, University of Alberta, Edmonton, AB Canada; 3 Brain-Body Institute and Department of Medicine, McMaster University, Hamilton, ON Canada

## Abstract

**Background:**

Allergic sensitization to aeroallergens develops in response to mucosal exposure to these allergens. Allergic sensitization may lead to the development of asthma, which is characterized by chronic airway inflammation. The objective of this study is to describe in detail a model of mucosal exposure to cockroach allergens in the absence of an exogenous adjuvant.

**Methods:**

Cockroach extract (CE) was administered to mice intranasally (i.n.) daily for 5 days, and 5 days later mice were challenged with CE for 4 consecutive days. A second group received CE i.n. for 3 weeks. Airway hyperresponsiveness (AHR) was assessed 24 h after the last allergen exposure. Allergic airway inflammation was assessed by BAL and lung histology 48 h after the last allergen exposure. Antigen-specific antibodies were assessed in serum. Lungs were excised from mice from measurement of cytokines and chemokines in whole lung lysate.

**Results:**

Mucosal exposure of Balb/c mice to cockroach extract induced airway eosinophilic inflammation, AHR and cockroach-specific IgG1; however, AHR to methacholine was absent in the long term group. Lung histology showed patchy, multicentric damage with inflammatory infiltrates at the airways in both groups. Lungs from mice from the short term group showed increased IL-4, CCL11, CXCL1 and CCL2 protein levels. IL4 and CXCL1 were also increased in the BAL of cockroach-sensitized mice in the short-term protocol.

**Conclusions:**

Mucosal exposure to cockroach extract in the absence of adjuvant induces allergic airway sensitization characterized by AHR, the presence of Th2 cytokines in the lung and eosinophils in the airways.

## Background

Atopy and allergic diseases affect more than 30% of the population worldwide. A study in 10 European countries showed that if we only take into account respiratory allergic conditions they still have a prevalence between 11.7% and 36.6% [1]. The economic burden of these diseases is also very high [2]. Despite intense efforts over the last 3 decades, the mechanisms controlling the development of allergic sensitization are still poorly understood. Animal models have been shown to be invaluable in allowing us to understand the pathogenesis of allergic conditions, especially asthma.

Animal models of asthma have a number of limitations, including the physiological relevance of the allergen used and potential differences between humans and animals in the development of allergic immune responses. There is also controversy regarding the utility of murine models of asthma as predictors of the response of human asthma to therapeutics [3]. Some of these difficulties arise from inadequacies of the murine models we use. However, other differences come from the way we use murine models and the kinds of responses we expect them to predict.

These limitations make it imperative that we use more than one model of asthma to understand specific questions and that we tailor the model to the question asked. The development of new murine models, especially of models that use clinically relevant allergens, may allow us to overcome some of these problems. In addition, models using relevant allergens may be proven to be invaluable for our understanding of the development of allergic sensitization. To this end researchers have developed a number of different models in mice.

Cockroach allergens are important sensitizing agents that may contain significant allergenic activity and are an important cause of asthma exacerbations in many parts of the World [4]. Several studies indicate that early life exposure to cockroach allergens leads to the development of specific allergic sensitization to cockroaches. Childhood sensitization has been associated with an increased risk for persistent asthma and bronchial hyper-responsiveness and with a greater loss of lung function [5]. In this regard, a strong relationship between indoor allergic sensitization and exacerbation of asthma symptoms has been demonstrated for cockroach [6]. Although most of the common cockroach allergens are not serine proteinases, we inhale these allergens along with a variety of cockroach-derived proteins with serine proteinase activity. These cockroach-derived proteins we inhale are from several sources, including cockroach saliva, feces, cast skins, debris and dead bodies [7].

Here we present a model that uses a physiological allergen, cockroach allergens present in whole body cockroach extracts, to cause allergic sensitization, and also a physiological airway mucosal route of sensitization. The objective of the study was to describe in more detail than what is available in the literature the characteristics of allergic airway inflammation and airway hyperresponsiveness that develop as a response to mucosal sensitization to cockroach allergens.

## Methods

### Animals

Male BALB/c mice and C57Bl/6 mice (6-8 weeks old) were purchased from Charles River Laboratories. All mice were housed in virus- and Ab-free conditions and maintained on a 12-h light-dark cycle. All experiments described in this manuscript were approved by the University of Alberta Health Sciences Laboratory Animal Ethics Committee (Edmonton, AB, Canada).

### Intranasal administration of whole cockroach extract (CE)

**Figure 1:**
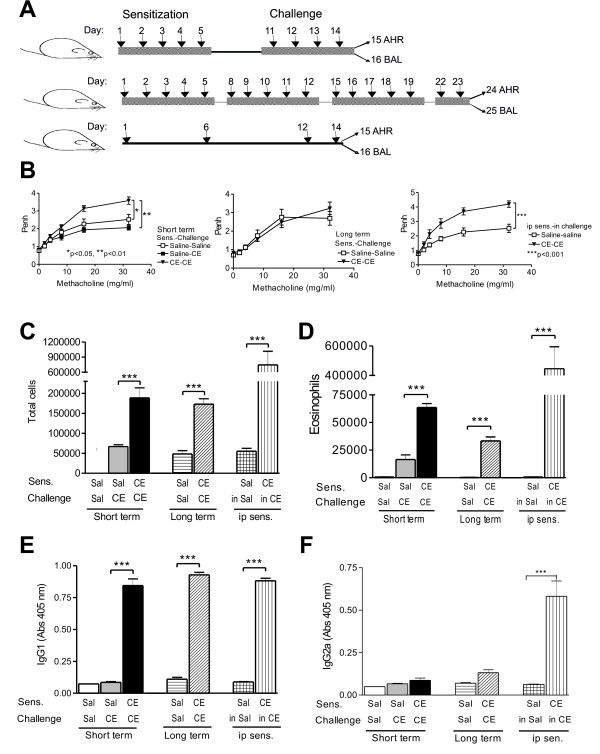
**Sensitization of Balb/c mice to CE allergens**. A. Models of mucosal and intraperitoneal sensitization to CE. B. AHR (Penh) was assessed 24 h after the last challenge for mice sensitized using the short-term (saline-saline n = 5; saline-CE n = 5; CE-CE n = 5; left graph) long-term (saline-saline n = 6; CE-CE n = 13; middle graph) and intraperitoneal (saline-saline n = 5; CE-CE n = 8; right graph) models. Allergic airway inflammation was measured 24 h after AHR by assessing the total cell numbers in the BAL fluid for the (C) short-term (saline-saline n = 8; saline-CE n = 8; CE-CE n = 8), long-term (saline-saline n = 6; CE-CE n = 14) and intraperitoneal models (saline-saline n = 7; CE-CE n = 7) as well as total eosinophils in the BAL fluid (D) for the short term (saline-saline n = 8; saline-CE n = 8; CE-CE n = 8), long-term (saline-saline n = 6; CE-CE n = 14) and intraperitoneal models (saline-saline n = 7; CE-CE n = 7). The serum from these mice was analyzed using ELISA for the presence of cockroach-specific IgG1 antibodies (E) and cockroach-specific IgG2a antibodies (F) for the short term (saline-saline n = 3; saline-CE n = 5; CE-CE n = 3), long-term (saline-saline n = 5; CE-CE n = 12) and intraperitoneal models (saline-saline n = 7; CE-CE n = 7). Values shown in B-F are means ± SEM.

Lyophilized frozen whole body extracts from German cockroach (*Blattella germanica*) were obtained from Greer Laboratories, (Lenoir, NC, USA) and were resuspended in sterile normal saline and stored at 4°C. Following light anesthesia with ketamine (75 mg/kg) and acepromazine (2.5 mg/kg), mice were given 50 μg of whole cockroach extract dissolved in 25 μl of sterile normal saline intranasally (i.n.) using the short-term and long term protocols shown in Figure 1A. Control mice were given 25 μl of sterile normal saline at the same time points. Other mice were sensitized with an i.p. injection of 50 μg of CE and 4 mg of Al(OH)_3 _in 0.5 ml of 0.9% sterile saline and then challenged with CE i.n., as shown in Figure 1A. In this case an i.p. injection of 0.5 ml of 0.9% sterile saline solution without CE was used as a negative control.

### Evaluation of AHR and allergic airway inflammation

Twenty-four hours after the final i.n. CE administration, we measured enhanced pause (Penh) to increasing doses of methacholine by non-invasive whole-body plethysmography (Buxco Electronics, Wilmington, NC, USA) to determine AHR as described [8]. Mice were euthanized 48 h after the last challenge for blood collection by cardiac puncture and lung lavage. The lungs were lavaged five times with 1 ml of isotonic phosphate-buffered saline pH 7.4 and 5 ml of this bronchoalveolar lavage (BAL) fluid was collected. Lungs were then removed and kept frozen until being processed for protein analysis. Protein analysis of lung lysates and BAL fluid was performed by Eve Technologies Corporation (Calgary, Canada) using a multiplex assay based on color-coded polystyrene beads.

The BAL fluid was centrifuged at 300 g for 5 min. Total cells were counted and then cytospins of 5000 cells were prepared and stained with Diff-Quick (Fisher Scientific Co, Kalamazoo, MI, USA). Airway inflammation was assessed by counting the number of inflammatory cells in the BAL fluid as previously described [9]. The cellular composition of bronchoalveolar lavage fluid was also assessed by FACS analysis. BAL cells were stained with anti-CD3, CD11c, CD19, MHCII, and CCR3 antibodies (BD Pharmingen, San Diego, CA). Data were acquired using a FACS Canto (Becton Dickinson, Oakville, Canada) and analyzed using FlowJo software (TreeStar, Ashland, OR). Lymphocytes were identified as Forward Scatter low/Side Scatter low cells expressing CD3 (T cells) or CD19 (B cells). Granulocytes were recognized as Side Scatter high cells and eosinophils were defined as Side Scatter high cells that express the eotaxin receptor CCR3 and intermediate levels of CD11c but very low to undetectable levels of MHCII, CD19 and CD3. Neutrophils were detected in a similar scatter profile as eosinophils but lacked CCR3 expression. Dendritic cells were identified as CD3^-^CD19^- ^cells that express high levels of MHCII and CD11c.

### Detection of cockroach specific IgG1 and IgG2a

Cockroach-specific IgG1 and IgG2a in mouse serum were measured by ELISA. Briefly, CE was incubated overnight at 4°C in 96-well NUNC MaxiSorp plates (Corning Costar Corp. NY, USA). Plates were then washed and blocked with PBS/10% FBS (GIBCO Invitrogen, Grand Islands, NY, USA), and mouse sera was added, and incubated for 3 h at 24°C. A 1:5,000 dilution of Biotin Rat anti-mouse-IgG1 (BD Pharmingen, Mississauga, ON, Canada) or Biotin Rat anti-mouse-IgG2a (BD Pharmingen) in PBS/10% FBS was added and plates were incubated for 1 h at 24°C, followed by the addition of a 1:1000 dilution (in PBS/10% FBS) of Horseradish peroxidase-conjugated streptavidin (Jackson ImmunoResearch Laboratories, Inc., Burlington, ON, Canada). TMB (BD Pharmingen) was added as a substrate, and allowed 20 min at 24°C to develop. The reaction was stopped by the addition of 2 N H_2_SO_4_, and the absorbance was measured at 405 nm on a Power Wave XS (BioTek Instruments Inc., Winooski, VT, USA) ELISA reader. Results are shown as absorbance units.

### Histological analysis

Animals were euthanized and the trachea intubated with a polyethylene catheter. Lungs were inflated with 10% neutral buffered formalin (Sigma-Aldrich, St. Louis MO, USA) for 10 min at a constant pressure of 20 cm water and then removed from the animal and placed in fresh 10% neutral buffered formalin for 24 h at 4°C before embedding in paraffin blocks. Sections (4 μm) were stained with H&E and PAS-D and analyzed using a Nikon Eclipse E-600 Microscope with 2X, 10X, 20X and 40X objectives. Pictures were taken using an 11-megapixel DXM 1200C Nikon Digital Camera.

### Statistical analysis

Values are expressed as "mean ± S.E.M". Statistical differences in the mean values among treatment groups were determined using a paired Student *t *test. In all cases, a value for p < 0.05 was considered statistically significant.

## Results

We have developed a model for mucosal sensitization of mice using whole body cockroach extract. Groups of mice received 50 μg of CE intranasally after anesthesia on 5 consecutive days, rested for 5 days and then received 50 μg of CE intranasally daily for 4 more days. Airway hyperresponsiveness (AHR) was assessed 24 h after the last allergen exposure and allergic airway inflammation was assessed 48 h after the last allergen exposure using BAL and lung histology (Figure 1A - short term protocol). To extend the duration of exposure to allergen we sensitized another group of mice with 50 μg of CE intranasally 5 days a week for 3 weeks, and for 2 consecutive days on the fourth week before evaluation for AHR and allergic airway inflammation as before (Figure 1A - long term protocol). Control groups of mice received saline on the same schedule. To compare our models with better-established models of parenteral sensitization in the presence of an adjuvant, a group of mice were sensitized with CE extract and Al(OH)_3 _i.p. and then challenged with CE i.n. (Figure 1A - i.p.).

**Figure 2:**
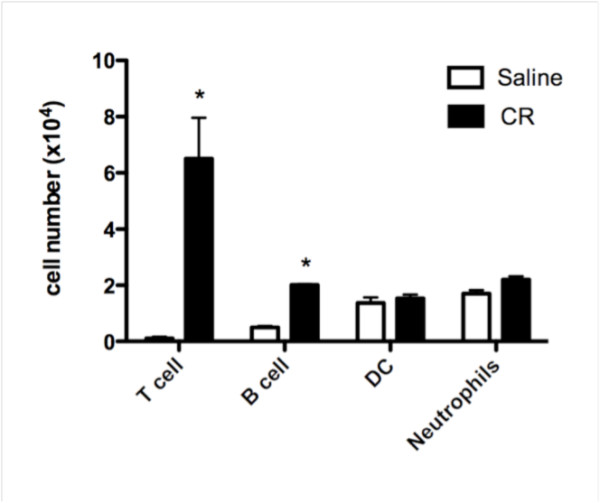
**Numbers of T lymphocytes, B lymphocytes, dendritic cells and neutrophils in the BAL fluid of mice sensitized and challenged with cockroach allergens using the short term protocol**. BAL fluid from cockroach sensitized and challenged mice and mice receiving only saline were analyzed by flow cytometry as described in Methods Section (n = 5 per group, * = p < 0.01).

Mice sensitized and challenged with CE using the short term protocol developed AHR (Figure 1B) and increased numbers of inflammatory cells (Figure 1C), primarily eosinophils (Figure 1D), in the airways, compared to mice who received saline only. FACS analysis also revealed an increase in T and B lymphocytes in mice that received CE in the short term protocol compared to mice that received saline, but no differences in the numbers of dendritic cells and neutrophils (Figure 2). Total lymphocytes in the BAL also increased in the mice that received CE in the long term protocol (from 416 ± 126 cells per mouse in mice receiving saline to 1807 ± 320 cells per mouse in mice receiving CE, n = 9, p < 0.02), but CE again had no effect on neutrophil numbers. Mice exposed to CE in the long term protocol showed less pronounced allergic responses. There was no evidence of AHR in the long term protocol and the numbers of eosinophils were diminished compared to the short term protocol (Figure 1). In both the short and long term protocols allergic airway inflammation was lower than what was seen with i.p. sensitization to CE in the presence of Al(OH)_3 _(Figure 1C and 1D).

We also evaluated the presence of antigen-specific antibodies. Mice receiving CE developed cockroach-specific IgG1 (Figure 1E), the levels of which were not different between the short term, long term and i.p. models. Mice that underwent the short term and long term protocols had no evidence of increased cockroach-specific IgG2a, while mice undergoing the i.p. protocol did develop antigen-specific IgG2a (Figure 1F).

**Figure 3:**
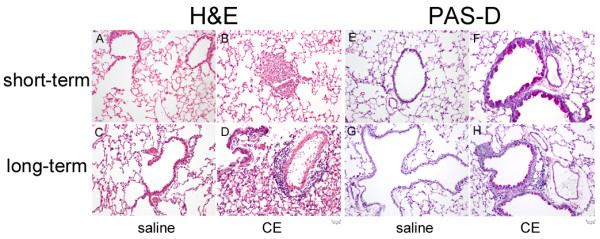
**Histological analysis of mice sensitized to cockroach allergens**. Representative pictures from mice sensitized under the short term protocol (A, B, E and F) and long-term protocol (C, D, G and H) are shown. A-D H&E for mice sensitized and challenged with saline (A and C) or CE (B and D). E-G PAS-D staining for mice sensitized and challenged with saline (E and G) or CE (F and H).

We performed histological analysis of lungs from mice sensitized and challenged with CE using both the short-term and the long-term models (Figure 3). Hematoxylin and eosin (H&E) stained sections showed normal lung architecture without inflammation in the airways or interstitium of mice sensitized with saline in the short-term (Figure 3A) and long-term (Figure 3C) models. Histological sections from mice sensitized and challenged with CE in the short-term model showed lung parenchyma with rare foci of inflammation, which in the case shown is partially destroying a small airway. Surrounding alveolar parenchyma was generally normal (Figure 3B). Mice sensitized and challenged with CE in the long-term model showed patchy, perivascular inflammation without large aggregates of inflammatory cells and mild edema (Figure 3D). Airways and surrounding alveolar parenchyma were largely normal. PAS with diastase (PAS-D) stained sections from mice sensitized and challenged with saline showed no evidence of mucin-containing epithelial cells in the airways (PAS-D negative sections) (Figure 3E and 3G). PAS-D stained sections from short-term (Figure 3F) and long-term (Figure 3H) CE sensitized mice showed numerous mucin containing epithelial cells (goblet cells) in the airways.

**Figure 4:**
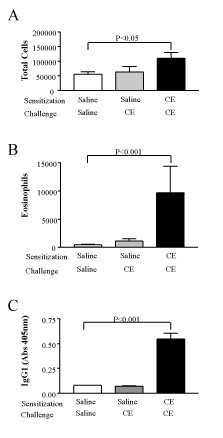
**Sensitization of C57Bl/6 mice to CE allergens**. AHR (Penh) was assessed 24 h after the last challenge (A) (saline-CE n = 12; CE-CE n = 16). Allergic airway inflammation was assessed 24 h after AHR using total cell numbers (B) and total eosinophils (C) present in BAL fluid (saline-CE n = 12; CE-CE n = 16). The serum from these mice was analyzed using ELISA for the presence of cockroach-specific IgG1 antibodies (D) (saline-CE n = 3; CE-CE n = 3). Values shown are means ± SEM.

We also sensitized C57Bl/6 mice using the short term protocol. Because we detected a large difference between mice receiving saline in the sensitization phase and CE in the challenge phase vs. mice receiving CE in both phases in Balb/c mice, we used only these two groups for the C57Bl/6 experiments. Mice that received CE in both phases showed increased total cells (Figure 4A) and eosinophils (Figure 4B) in BAL fluid, and increased cockroach-specific IgG1 (Figure 4C) compared to mice that received saline during the sensitization phase and CE during the challenge phase. These mice also showed evidence of AHR (data not shown). This indicates that the cockroach extract has similar effects in Balb/c and C57Bl/6 mice and that both strains can be sensitized to CE intranasally.

**Figure 5:**
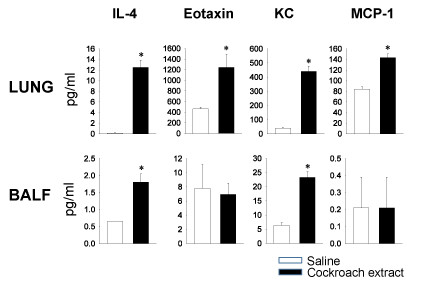
**Cytokines and chemokines in lung tissue and BAL fluid of cockroach-sensitized and challenged mice**. Levels of IL-4, CCL11 (eotaxin), CXCL1 (KC) and CCL2 (MCP-1) in lung lysates (upper panels) and BAL (lower panels) from mice challenged with saline or CE (n = 5, *p < 0.05).

We then studied the presence of cytokines and chemokines in the lung tissue and airways of cockroach-sensitized and challenged mice. Lungs were removed from mice undergoing the short term protocol and then lyzed to measure cytokines and chemokines using a multiplex assay based on color-coded polystyrene beads. IL-4, CCL11 (eotaxin), CXCL1 (KC) and CCL2 (MCP-1) were increased in the lungs of sensitized and challenged mice (Figure 5). An increase in IL-4 and CXCL1 was also seen in the BAL fluid of these mice, but CCL11 and CCL2 were not increased in the BAL fluid (Figure 5). In all cases there were significantly lower levels of the cytokines in the BAL fluid compared to the lung lysate. In general the protein assay was not sensitive enough to pick up most of the cytokines and chemokines analyzed in BAL fluid and for most of those that could be measured in the BAL fluid there was no difference between naïve and allergen sensitized and challenged mice (data not shown).

**Figure 6:**
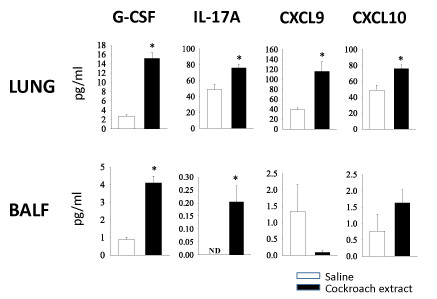
**G-CSF, IL-17A, CXCL9 and CXCL10 levels in lung tissue and BAL fluid of cockroach-sensitized and challenged mice**. Levels of these cytokines and chemokines in lung lysates (upper panels) and BAL (lower panels) from mice challenged with saline or CE (n = 5, *p < 0.05).

A number of other cytokines/chemokines were also significantly increased in the lung tissue of mice that were sensitized and challenged with CE. IL-5 and IL-13 were increased, as expected, but IL-12p40, GM-CSF, CCL3 (MIP-1α), CCL4 (MIP-1β) were also increased (data not shown). Also G-CSF, IL-17A, CXCL9 (MIG), and CXCL10 (IP10) were elevated in the lung tissue (Figure 6). G-CSF and IL-17A were also increased in the BAL fluid of these mice (although the levels were significantly lower than the levels in the lung tissue), but CXCL9 and CXCL10 were not increased in the BAL fluid (Figure 6). However, IL-10, IFNγ, RANTES and VEGF were not increased following sensitization and challenge with CE.

## Discussion

In this manuscript we present a detailed analysis of the airway inflammation present in a model of mucosal sensitization to cockroach allergens. Mice sensitized to cockroach through the intranasal route in the absence of an adjuvant developed all the expected characteristics of asthma; they developed AHR, eosinophilic airway inflammation and allergen-specific IgG1 antibodies after exposure to allergens over a period of 2 weeks. These mice also showed increased levels of Th2 cytokines and a number of chemokines in the lung tissue. Mucosal exposure to CE led to allergic inflammation in both Balb/c and C57Bl/6 mice, although the latter had lower numbers of eosinophils accumulating in the airways. Mice exposed to CE for longer periods, 17 intranasal exposures over 31/2 weeks, showed lower AHR and less eosinophils in the BAL compared to mice treated with the short term model. Histological evaluation of the short and long term models showed changes compatible with allergic airway inflammation. The changes seen in the lungs stained with H&E were quite mild compared to other murine models of asthma. CE induced inflammation in both the short and long term models compared to non-sensitized mice. The inflammation seen in the long term model was more diffuse with smaller aggregates of inflammatory cells than what was seen in the short term model. PAS-D staining of the lungs of CE sensitized and challenged mice showed increased goblet cells in the airways in both models compared to non-sensitized mice. The exact reason for decreased levels of inflammation in the long term protocol are not known. Other groups using various models have shown that longer exposure to allergens can lead to tolerance and therefore have decreased levels of inflammation compared to the allergic inflammation seen following shorter exposure periods [10,11]. It is possible that a similar effect is seen in our model.

Allergic sensitization to cockroach allergens has been associated with severity of asthma in inner cities of USA. Animal models using these allergens may allow us to better understand the role of these antigens in allergy and asthma. Cockroach allergens have been used previously in murine models of allergic sensitization [12-14], but those studies used an intraperitoneal injection of cockroach in the presence of an adjuvant to sensitize mice. Recently our laboratory [15] and others [16,17] have developed models of mucosal sensitization to cockroach allergens without any added adjuvant, similar to models using house dust mite allergens. Our model presented here and in our previous work [15] differs from that used by Page and colleagues [16,17] because we used whole cockroach body extract, rather than cockroach frass, to induce allergic sensitization. However, the two models have many similarities. Our aim here was to describe this model in more detail and analyze various aspects of the inflammatory environment induced by mucosal sensitization to cockroach allergens.

Our data indicate that i.n. sensitization generates a more Th2-like IgG response (increased antigen-specific IgG1, but not IgG2a) compared to murine models that use i.p. sensitization (both IgG subtypes were increased). In accordance with our data, Phipps et al., were unable to detect allergen-specific IgG2a in i.n. sensitized mice [18], while Ng et al., only detected allergen-specific-IgG2a in the serum of mice when an allergen was given i.n. with a strong Th1 polarizing stimulus [19]. These data indicate that mucosal sensitization to cockroach extract leads to a more Th2 skewed model of airway inflammation compared to mice sensitized intraperitoneally in the presence of an adjuvant. Our model therefore, induced a strong allergic phenotype, which supports the validity and value of this model to study mechanisms of allergic sensitization.

We also analyzed the cytokines and chemokines present in the lungs of mice sensitized and challenged with our short term protocol. We identified increased levels of Th2 cytokines (IL-4, IL-5 and IL-13) as would be expected, as well as increased levels of IL-17A and G-CSF. Increases in IL-17A and G-CSF have been shown previously in a slightly different model of sensitization to cockroach allergens, although in that case the increase was seen in cultured lung cells stimulated with conA [16,17].

We also identified the presence of CXCL1 (KC) in the lungs of cockroach-sensitized and challenged mice. CXCL1 (KC) is a chemotactic factor for neutrophils. Our model does not have a high number of neutrophils recruited to the lung. However, it has been shown in a model of OVA-induced allergic airway inflammation that CXCL1 (KC) also recruits endothelial progenitors to the lung [20]. The same molecule has been shown to be present in human induced sputum 24 h after allergen challenge and correlates with endothelial progenitor recruitment in the airways [21]. Furthermore, a molecular phenotype characterized by the presence of CXCL1, RANTES, IFNγ, IL-12 and IL-10 separated children with severe asthma from those with moderate asthma [22]. Further work on the presence and role of CXCL1 (KC) in murine models of allergic inflammation may allow us to better understand the pathogenesis of disease and vascular remodeling during asthma.

Cockroach is a complex mix of many proteins including serine proteinases. A number of common environmental allergens, including cockroach allergens, have enzymatic activity, which may skew the immune response toward the Th2 phenotype [23] and mediate, or at least participate in, the development of allergic sensitization. We have published evidence that the serine proteinase activity of cockroach allergens and their ability to activate Proteinase-Activated Receptor-2 (PAR-2) may be an important determinant of the ability to induce allergic sensitization [15]. Developing a model of allergic sensitization using cockroach extract will allow us to dissect the complexity of the extract and to identify the ability of individual antigens to induce allergic sensitization or to function as effective allergens. For example, one cockroach allergen, Per a 10, has been cloned and its proteolytic activity has been shown to be important for the development of allergic inflammation and AHR [24]. Availability of many different models of mucosal sensitization to be used in comparative studies may also allow us to better understand the basic mechanisms that participate in allergic sensitization and allergic airway inflammation. There is already evidence that different allergens induce different activation pathways in the airways in murine models [25] and comparing more models may expand our knowledge in this field.

These studies also allow us to better understand the regulation of the mucosal immune system in the lung. Mucosal immunology has been, for decades, an area of study in gastrointestinal diseases and has allowed an understanding of many facets of the immune system [26,27]. Translation of these studies into the airways will allow us to obtain a similar understanding of the airway mucosal immune responses.

## Conclusions

In conclusion, we have presented a detailed analysis of a model of allergic sensitization using mucosal exposure to cockroach allergens, which is functional in both Balb/c and C57Bl/6 mice. This model may allow us to better understand the role of cockroach allergens in allergic disease and in the inner city asthma epidemic.

## List of Abbreviations Used

AHR: airway hyperresponsiveness; BAL: bronchoalveolar lavage; CE: cockroach extract; H&E: hematoxylin and eosin; i.n.: intranasal; i.p.: intraperitoneal; PAS-D: periodic acid shift with diastase

## Competing interests

The authors declare that they have no competing interests.

## Authors' contributions

NGA carried out most of the animal studies, participated in the study design and drafted the manuscript. MA carried out animal studies and participated in the study design. LP performed all histological analysis and prepared the histological pictures. MA performed the cytokine/chemokine analysis. CD participated in the studies analyzing airway inflammation and helped draft the manuscript. KK performed the flow cytometry studies and contributed in study design. PF participated in the study design and helped draft the manuscript. HV participated in the study design and helped draft the manuscript. All authors read and approved the final manuscript.
